# Protective effects of nuclear factor erythroid 2-related factor 2 on whole body heat stress-induced oxidative damage in the mouse testis

**DOI:** 10.1186/1477-7827-11-23

**Published:** 2013-03-21

**Authors:** Yansen Li, Yi Huang, Yuanguo Piao, Kentaro Nagaoka, Gen Watanabe, Kazuyoshi Taya, ChunMei Li

**Affiliations:** 1 College of Animal Science and Technology, Nanjing Agricultural University, 1 Weigang Road, Nanjing, 210095, P.R. China; 2 Laboratory of Veterinary Physiology, Cooperative Department of Veterinary Medicine, Faculty of Agriculture, Tokyo University of Agriculture and Technology, Tokyo, Japan; 3 Department of Basic Veterinary Science, The United Graduate School of Veterinary Sciences, Gifu University, Gifu, Japan

**Keywords:** Whole body heat stress, Nrf2, Oxidative stress, Germ cell, Leydig cell, Mice

## Abstract

**Background:**

Whole body heat stress had detrimental effect on male reproductive function. It's known that the nuclear factor erythroid 2-related factor 2 (Nrf2) activates expression of cytoprotective genes to enable cell adaptation to protect against oxidative stress. However, it’s still unclear about the exactly effects of Nrf2 on the testis. Here, we investigate the protective effect of Nrf2 on whole body heat stress-induced oxidative damage in mouse testis.

**Methods:**

Male mice were exposed to the elevated ambient temperature (42°C) daily for 2 h. During the period of twelve consecutive days, mice were sacrificed on days 1, 2, 4, 8 and 12 immediately following heat exposure. Testes weight, enzymatic antioxidant activities and concentrations of malondialdehyde (MDA) and glutathione (GSH) in the testes were determined and immunohistochemical detection of Nrf2 protein and mRNA expression of Nrf2-regulated genes were analyzed to assess the status of Nrf2-antioxidant system.

**Results:**

Heat-exposed mice presented significant increases in rectal, scrotal surface and body surface temperature. The concentrations of cortisol and testosterone in serum fluctuated with the number of exposed days. There were significant decrease in testes weight and relative testes weight on day 12 compared with those on other days, but significant increases in catalase (CAT) activity on day 1 and GSH level on day 4 compared with control group. The activities of total superoxide dismutase (T-SOD) and copper-zinc SOD (CuZn-SOD) increased significantly on days 8 and 12. Moreover, prominent nuclear accumulation of Nrf2 protein was observed in Leydig cells on day 2, accompanying with up-regulated mRNA levels of Nrf2-regulated genes such as *Nrf2*, heme oxygenase 1 (*HO-1*), γ-Glutamylcysteine synthetase (*GCLC*) and NAD (P) H: quinone oxidoreductase 1 (*NQO1*)) in heat-treated groups.

**Conclusions:**

These results suggest that Nrf2 displayed nuclear accumulation and protective activity in the process of heat treated-induced oxidative stress in mouse testes, indicating that Nrf2 might be a potential target for new drugs designed to protect germ cell and Leydig cell from oxidative stress.

## Background

In most mammals, the testes normally complete descent into a scrotum before birth to provide a lower temperature for spermatogenesis [1]. If descent does not occur and testis remains in abdomen, it’s described as the cryptorchidism which present invariably sterile and spermatogenesis does not begin until testis is surgically moved into the scrotum [2]. Although testes suspend in a scrotum outside the body cavity, spermatogenesis is still disturbed by exposure to high ambient temperature via weakening the ability of thermoregulatory system and inducing increased temperature in testis. Air temperature above 40°C caused scrotal temperature to rise to the value of deep body temperature in rams and rats exposed to an environment of 35°C also exhibited an higher intra-scrotal temperature closed to the core body temperature [3,4]. In addition, whole body heat exposure resulted in high scrotal temperature and poor semen quality in boars, mice and human [5-7]. The correction or prevention of whole body heat stress-induced sterility is therefore a problem of major concern.

Nuclear factor erythroid 2-related factor 2 (Nrf2), a Cap'n'Collar basic leucine zipper transcription factor, plays an important role in preventing the development of oxidative stress through up-regulation of the Nrf2-related antioxidants [8,9]. Under homeostatic conditions, the synthetic Nrf2 is captured by Kelch-like ECH-associated protein 1 (Keap1) and constitutively degraded via the ubiquitin-proteasome pathway in cytoplasm [10]. The presence of oxidative or electrophilic stresses can modify cysteine residues on Keap1, thereby inactivating it [11]. As a result, Nrf2 is stabilized and exported into the nuclei to stimulate the transcription of antioxidant genes through heterodimerization with a small musculoaponeurotic fibrosarcoma (Maf) protein and binding to the antioxidant response element (ARE) sequence [10]. The up-regulated antioxidants reverse serve to scavenge the oxidative and electrophilic stressors.

A recent study found that scrotal heat stress induced severe oxidative stress in mouse testes, which consequently caused germ cell death [12]. Moreover, Nrf2-knockout mouse presented an oxidative disruption in spermatogenesis [13]. However, it's still unclear whether Nrf2 can protect germ cell against oxidative stress in testes via Nrf2-antioxidant pathway. In this study, male mice were exposed to the room temperature of 42°C to investigate the impact of whole body heat on the temperature parameters, relative testes weight, levels of MDA and GSH, as well as the activities of enzymatic antioxidants. We hypothesized that Nrf2 accumulated in nucleus and regulated the Nrf2-regulated genes against heat-induced oxidative stress.

## Methods

### Animals

Male adult Institute of Cancer Research (ICR) mice, 8 weeks old, were purchased from Nanjing Qinglongshan Experimental Animal Center. The animals were provided with food and water *ad libitum* and were maintained on a 12 h light–dark cycle in a controlled temperature (25°C) and humidity (50% ± 5%). The animal procedures were approved by the Institutional Animal Care and Use Committee of Nanjing Agricultural University.

### Heat treatment

**Figure 1:**
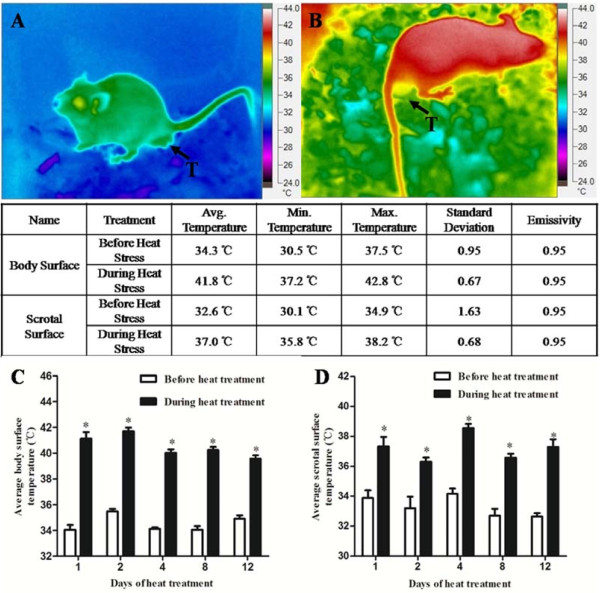
**Body and scrotal surface temperatures of mice before and during heat treatment.** The table showed the body and scrotal surface temperatures of each mouse before (**A**) and during (**B**) heat treatment, as measured one time using a thermal imaging camera. (**C**) Mouse body surface temperatures were measured before and during heat treatment on days 1, 2, 4, 8 and 12. Average body surface temperature was achieved by counting the average of body surface temperatures of five mice, paired t-test, body surface temperature during treatment vs. before treatment, * P < 0.05. **(D**) Mice scrotal surface temperatures were measured before and during heat treatment on days 1, 2, 4, 8 and 12. Average scrotal surface temperature was achieved by counting the average of scrotal surface temperatures of five mice, scrotal surface temperature during treatment vs. before treatment, * P < 0.05. **T**, testis. Each bar represents the mean ± SEM (n = 5).

To investigate the effect of the whole body heat stress on testicular oxidative stress and Nrf2-antioxidant system, all mice except controls (0 day) were additionally exposed to 42°C between 11:00 h and 13:00 h daily for twelve consecutive days. In the conditions of mice total recovery after heat treatment and feasible construction of chronic heat model, whole body heat temperature of 42°C for 2 h was chosen according to previous reports [3,14]. Mouse body temperature was recorded daily before and during heat exposure using a thermistor probe connected to a digital thermometer (DM6801A, SAMPO, Shenzhen, China). Scrotal and body surface temperature were also recorded daily before and during heat exposure using a thermal imaging camera (TiR27, FLUKE Corporation, Washington, USA). As shown in Figure 1, individual scrotal surface temperature was defined as the average value of whole testis surface temperature and individual body surface temperature was defined as the average value of temperature of whole body including head, ears, trunk body, legs and tail. Both individual scrotal and body temperatures were calculated using Fluke Smart View 3.1 imager software. On the 1st, 2nd, 4th, 8th and 12th days, five mice from heated group were sacrificed immediately following heat exposure. The mice from control group and heat treated group on the 1st day were sacrificed at the same time. Blood samples were collected following sacrifice and centrifuged at 3,000 × g for 10 min. The isolated serum samples were stored at -80°C until determine. The testes were weighed and divided into two parts. Left testis was kept at -80°C for subsequent biochemical measure and RT-PCR. Right testis was immersed in a 4% paraformaldehyde solution for testicular histological analysis.

### Radioimmunoassay (RIA)

Concentrations of cortisol and testosterone in serum were measured by double-antibody RIAs using ^125^I-cortisol and ^125^I-testosterone Radioimmunoassay Kits (Beijing North Institute of Biological Technology, Beijing, China) according to manufacturer’s protocol. The intra- and inter-assay coefficients of variation were 6.1% and 9.2% for cortisol, and 6.9% and 7.2% for testosterone, respectively.

### MDA assay

Frozen testicular tissue was homogenized in ice-cold NaCl solution. The homogenate was centrifuged at 1,700 × g for 15 min at 4°C, and the MDA level in the supernatant was analyzed using the MDA assay kit (Jiancheng Bioengineering Institute, Nanjing, China) according to manufacture information. It was analyzed with thiobarbituric acid method by monitoring MDA-reactive products spectrophotometrically. The absorbance of the organic layer was measured at 532 nm. Data were expressed as nanomoles of MDA per milligram of testis protein.

### Analysis of antioxidant status

The samples as prepared above were used to analyze parameters of oxidative stress including GSH concentration and the activities of enzymatic antioxidants: T-SOD, CuZn-SOD, glutathione peroxidase (GPX), and catalase (CAT). Reduced GSH was determined by measuring absorbance at 412 nm, while T-SOD activity was measured at 560 nm. GPX activity was measured using a method detedts a complex with a maximal absorbance at 412 nm formed by the reaction of GSH remaining after the action of GPX with 5, 5'-dithiobis-2-nitrobenoic acid. One unit of GPX activity was defined as the decrease amount of 1 μmol/L of GSH per min (except the effect of non-enzymatic reaction) in system of enzymatic reaction of 1 mg protein per minute. CAT activity was measured as absorbance at 405 nm by using an assay based on the consumption of H_2_O_2_. The parameters above were determined using commercial kits (Jiancheng Bioengineering Ltd., Nanjing, China) according to the manufacturer's instructions.

### Immunohistochemistry

Following fixation of the testes, the fixed samples were passed through a graded series of ethanol and xylene solutions and embedded in paraffin wax. Serial sections (5 μm thick) cut from the paraffin-embedded tissues were transferred onto 3-aminopropyl-triethoxysilane (APES)-coated slides (S8441, MATSUNAMI, Tokyo, Japan). Sections were deparaffinized in xylene and rehydrated through a graded ethanol series. To increase epitope exposure, the sections were heated in sodium citrate buffer (0.01 M, pH 6.0) at 121°C for 15 min in an autoclave. After incubating in 3% H_2_O_2_ in methanol (v/v) at 32°C for 30 min, the sections were blocked with normal goat serum for 1 h and then incubated overnight at 4°C with rabbit polyclonal antibodies specific for Nrf2 (Santa Cruz Biotechnology, California, USA), diluted 1: 200 in phosphate-buffered saline (PBS, 0.01 M, pH 7.2). Control sections were incubated with blocking serum alone. The specific protein immunoreactivity was visualized with the VECTASTAIN ABC Kit (Histofine, Tokyo, Japan) and a DAB kit (Histofine, Tokyo, Japan). To identify structural features in tissue and assess cell morphology, the sections were counterstained with hematoxylin and mounted with coverslips. Immunostaining was evaluated digicalized images snaped with an Olympus camera.

### RNA extraction and quantitative real-time PCR

Total RNA was isolated from frozen testes using TRIzol reagent (Invitrogen, Carlsbad, CA, US) and treated with DNase І (RNase-free) (TaKaRa, Dalian, China) to remove genomic DNA. The total RNA concentration and purity were determined by a spectrophotometer (SMOIF, Shanghai, China). For each sample, 5 μg of total RNA was reverse transcribed to cDNA with M-MLV reverse transcriptase (TaKaRa, Dalian, China) and oligonucleotide primers.

Targets genes and the housekeeping gene beta actin (*ACTB*) were quantified by real-time PCR on an ABI 7300 system using a commercial kit (SYBR Premix Ex Taq, TaKaRa, Dalian, China). The gene-specific primers were designed based on the corresponding mRNA sequences with Primer Version 5.0 (Table 1). PCR reactions (consisting of SYBR Premix Ex Taq, ROX Reference Dye, 200 nM primer, and 100 ng cDNA template) were run in triplicates in a 20-μl total reaction volume. The amplification conditions were as follows: DNA polymerase activation at 95°C for 30 sec, followed by 42 amplification cycles of denaturation at 95°C for 5 sec, annealing at 58°C for 30 sec, and extension at 72°C for 30 sec. The specificity of the PCR product was verified with a melting curve and by agarose gel electrophoresis. The relative mRNA concentration was calculated using the 2^-ΔΔCt^ method [15]. All samples were measured in triplicate. The values were normalized using *ACTB* as the endogenous standard.

**Table 1:** Primers used for real-time PCR

**Gene (abbreviation)**	**GenBank accession no.**	**Sequence (5' → 3')**	**Length of DNA product (bp)**
*β-actin (ACTB)*	NM_007393	F: CTGTCCCTGTATGCCTCTG	218
		R: ATGTCACGCACGATTTCC	
*Nuclear factor erythroid 2-related factor 2 (Nrf2)*	NM_010902	F: CAGTGCTCCTATGCGTGAA	109
		R: GCGGCTTGAATGTTTGTC	
*Heme oxygenase 1 (HO-1)*	NM_010442	F: ACAGATGGCGTCACTTCG	128
		R: TGAGGACCCACTGGAGGA	
*NAD(P)H: quinine oxidoreductase 1 (NQO1)*	NM_008706	F: CTTTAGGGTCGTCTTGGC	102
		R: CAATCAGGGCTCTTCTCG	
*γ-Glutamylcysteine synthetase (GCLC)*	NM_010295	F: GGATGATGCCAACGAGTC	180
		R: GTGAGCAGTACCACGAATA	

### Statistical analysis

The results are presented as the mean ± standard error of the mean (SEM). Temperature parameters were analyzed by paired t-tests to identify significant differences between mice before heat treatment and during heat treatment. Data from control and treated mice were analyzed by one-way analysis of variance (ANOVA) followed by Tukey's multiple comparison test. Statistical analysis was performed using GraphPad Prism Version 5.0 software program (GraphPad Software, San Diego, CA, USA). P < 0.05 was considered to indicate a statistically significant result.

## Results

### Assessment of heat treatment

**Figure 2:**
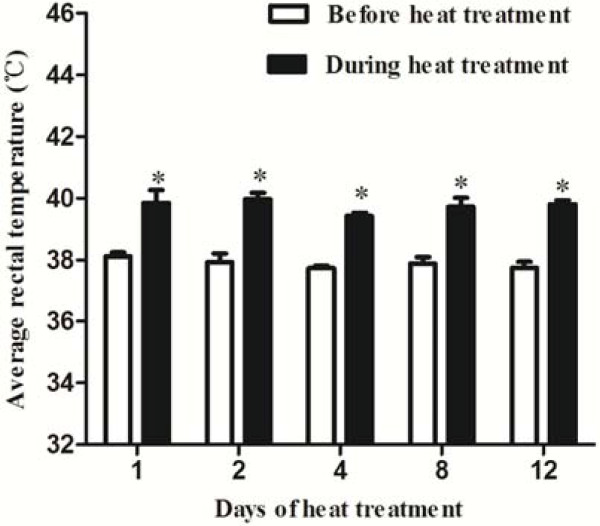
**Effects of heat treatment on average rectal temperature of mice before and during heat treatment.** Mouse rectal temperature were measured before and during heat treatment (42°C) using a digital thermometer on days 1, 2, 4, 8 and 12, paired t-test, rectal temperature during treatment vs. before treatment, * P < 0.05. Each bar represents the mean ± SEM (n = 5).

Mice rectal surface, scrotal surface and body surface temperatures were significantly increased after 2 h heat exposure at all five time points (Figure 1C, Figure 1D and Figure 2). Mice suffered heat stress when rectal temperature approached 40°C, characterized by an excessive drinking, increased respiratory and restlessness.

### Serum concentrations of cortisol and testosterone

**Figure 3:**
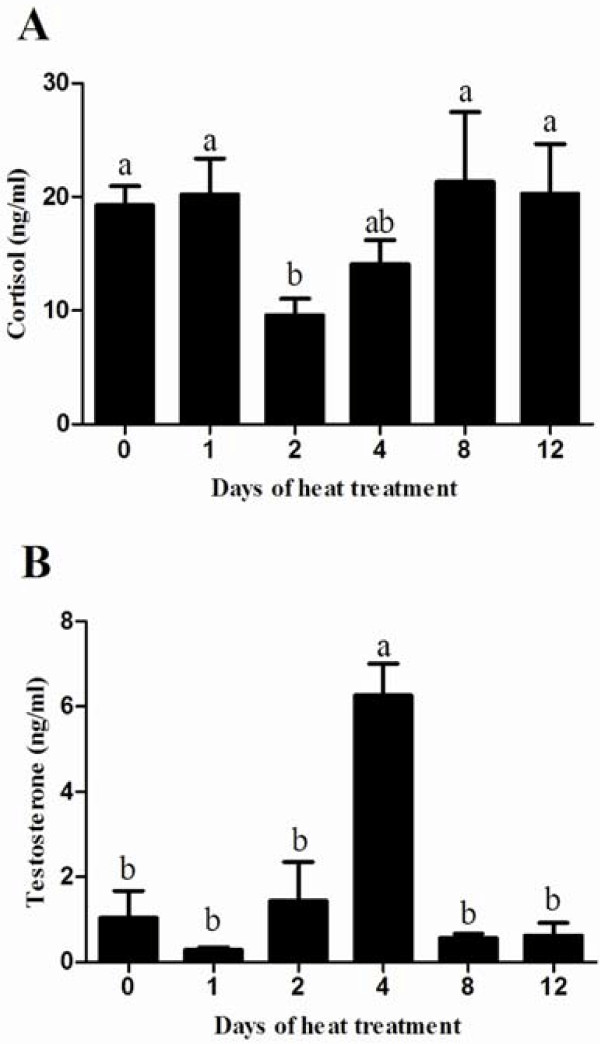
**Effects of heat treatment on serum cortisol and testosterone in mice.** The concentrations of cortisol (**A**) and testosterone (**B**) in serum were measured on days 0, 1, 2, 4, 8 and 12. Each bar represents the mean ± SEM (n = 5). Different superscripts above bar differ significantly between each other (P < 0.05).

Serum cortisol concentration was similar between each other, with exception of the significant decrease on day 2 comparing with control group (Figure 3A). Serum testosterone concentration in heat treated group increased significantly on day 4 and returned to normal on day 8 (Figure 3B).

### Body and testes weights

Upon heat treatment on day 8, body weight was significantly lower than those on days 1 and 2 (Table 2). Testes weight showed significant decrease on days 8 and 12 comparing with those on days 1, 2 and 4 respectively (Table 2). Relative testes weight was obtained by dividing testes weight by body weight. Relative testes weight, measured on day 12, was significantly lower than those on days 1, 2 and 4 respectively (Table 2). Alternatively, body and testes weight showed no marked change during period of heat treatment from day 1 to day 4 and decreased with exposure time during period of heat treatment from day 4 to day 12.

**Table 2:** Effects of heat treatment on body weight and testes weight in mice

**Treatment**	**Body weight (g)**	**Testes weight (mg)**	**Testis/body weight (mg/g)**
*0*	33.92 ± 0.36^ab^	204.0 ± 4.9^ab^	6.02 ± 1.06^ab^
*1*	36.65 ± 0.97^a^	243.4 ± 9.3^a^	6.66 ±3.53^a^
*2*	35.54 ± 1.36^a^	239.8 ± 11.6^a^	6.76 ± 1.08^a^
*4*	34.10 ± 1.31^ab^	247.2 ± 12.2^a^	7.28 ± 3.44^a^
*8*	29.98 ± 1.48^b^	173.4 ± 10.1^b^	5.84 ± 4.83^ab^
*12*	32.85 ± 1.03^ab^	167.4 ± 13.8^b^	5.09 ± 3.94^b^

Values are expressed as mean ± SEM (n = 5). Means in the same column with different superscripts were significantly different (P < 0.05).

### Oxidative stress parameter

**Figure 4:**
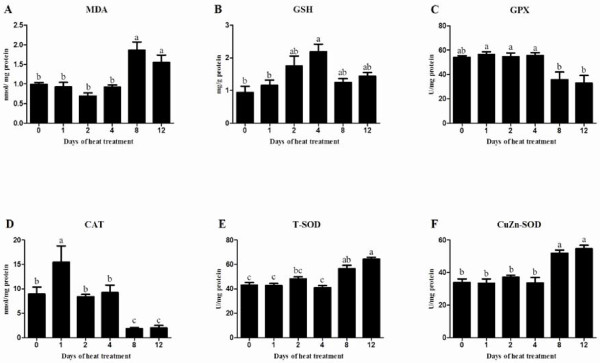
**Effects of heat treatment on oxidative stress parameters in the testes of mice.** The parameters (**A**, MDA; **B**, GSH; **C**, GPX; **D**, CAT; **E**, T-SOD and **F**, CuZn-SOD) were measured on days 0, 1, 2, 4, 8 and 12. Each bar represents the mean ± SEM (n = 5). Different superscripts above bar differ significantly between each other (P < 0.05).

There was no significant difference in MDA concentration, GSH amount and the activities of GPX, CAT, T-SOD and CuZn-SOD between control and heat-treated groups on days 1, 2 and 4, except for significant increased GSH amount on day 4 and increased CAT activity on day 1 (Figure 4). Compared with mice on day 4, mice on days 8 and 12 had lower GSH amount and less activities of GPX and CAT (P < 0.05), but higher MDA concentrations (P < 0.05) (Figure 4A, 4B, 4C and 4D). The activities of T-SOD and CuZn-SOD on days 8 and 12 were significantly higher than those in control and heat-treated groups on days 1 and 4 (Figure 4E and 4F).

### Heat stress-induced nuclear Nrf2 accumulation

**Figure 5:**
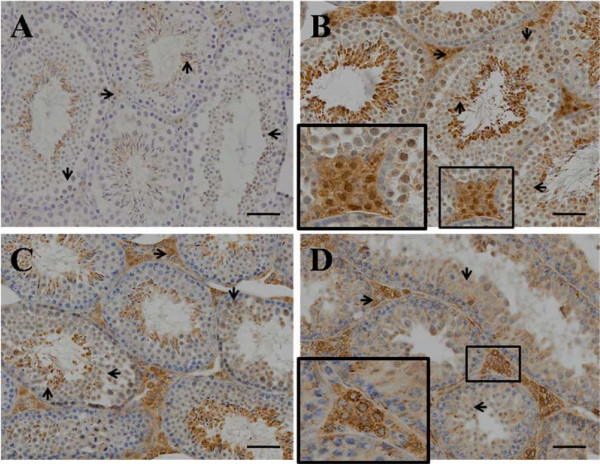
**Immunohistochemical detection of Nrf2 in the testes of mice.** Sections from heat-treated mice on 0 d (**A**), 2 d (**B**), 4 d (**C**) and 12 d (**D**) were stained with anti-body Nrf2. The immunohistochemical signals appear brown and the counterstained background appears blue in color. →, Leydig cell; ↓, spermatocyte; ←, round spermatid; ↑, enlongate spermatid. Bar = 100 μm.

Immunohistochemical analysis showed higher expression of Nrf2 protein in heated testes on days 2, 4 and 12 comparing with those in control group (Figure 5). Prominent nuclear accumulation of Nrf2 protein was observed in germ cells and Leydig cells in heated testes on days 2 and 4 (Figure 5B and 5C). Nuclear accumulation started to reduce in Leydig cells germ cells on day 12, especially for spermatogonia. However, fine granular cytoplasm localization of Nrf2 was also evident in Leydig cells on days 4 and 12 (Figure 5C and 5D). Furthermore, sections from heated testes on day 12 indicated that most enlongate spermatids and some round spermatids were lost (Figure 5C and 5D).

### Heat stress-induced Nrf2-regulated genes mRNA expression

**Figure 6:**
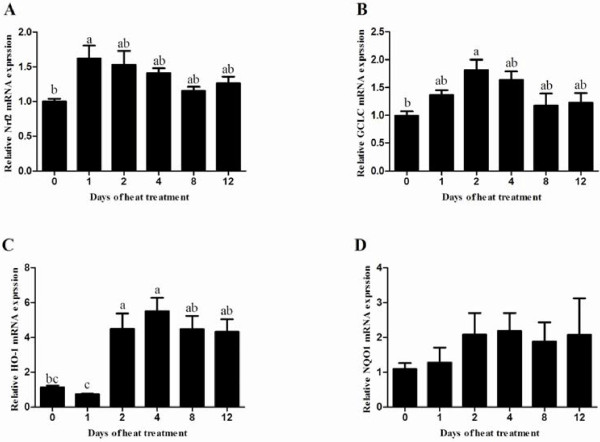
**Effects of heat treatment on mRNA expression of Nrf2-regulated genes in the testes of mice.** The mRNA levels of Nrf2-regulated genes (**A**, *Nrf2*; **B**, *GCLC*; **C**, *HO-1* and **D**, *NQO1*), as measured by real-time PCR. Values were normalized using *ACTB* as the endogenous standard. Each bar represents the mean ± SEM (n = 5). Different superscripts above bar differ significantly between each other (P < 0.05).

The mRNA transcript levels of *Nrf2* were slight decrease in heated testes comparing with those in control group, except for the level on day 4 significantly lower than those in control group (Figure 6B). The mRNA transcript levels of heme oxygenase 1 (*HO-1*) and γ-Glutamylcysteine synthetase (*GCLC*) were significantly up-regulated in heated testes with a *HO-1* peak on day 4 and a *GCLC* peak on day 2 respectively (Figure 6C and 6E). Both *HO-1* and *GCLC* mRNA levels had a tendency to return to baseline levels on day 12. There was no significant difference in NAD (P) H: quinone oxidoreductase 1 (*NQO1*) mRNA between each group with slight increase in heat treated groups at all five time points (Figure 6D).

## Discussion

The objective of the present study is to reveal the role of Nrf2 on suppressing the process of oxidative stress after whole body heat treatment, by which we might indentify new targets in diagnosed cases of oxidative stress-induced infertility diagnosis.

In mammals, heat stress is defined as an environment which acts to drive body temperature above set-point temperature [16]. If the ambient temperature exceeds 40°C, the scrotal temperature will rise to the values in the abdomen [3]. Heat stress can induce disruption in internal secretion system such as hypothalamic-pituitary-adrenal axis [17,18]. In this study, mice rectal temperature and body surface temperature were significantly increased after 2-h heat exposure (Figure 1 and 2). The fluctuant cortisol level in serum also confirmed the occurrence of heat stress induced by whole body heat exposure (Figure 3A). The scrotal surface temperature rose close to rectal temperature during heat treatment (Figure 1D). In addition, heat stress led to an elevated scrotal surface temperature, consistent with previous researches in boar and human [7].

Most mammal testes suspend in a scrotum outside the body cavity and are more susceptible to heat than other organs [2,16]. Scrotal temperature above the normal range can disrupt spermatogenesis and increase rates of sub or infertility in rodents, domestic animals, monkey and human [7,19-21]. In mice, acute or chronic testicular heat exposure led to a fall in testes weight [22,23]. Additionally, heat stress has been found to cause germ cell loss and poor quality semen [12,19]. In this study, both reduced testes weight and germ cell loss on day 12 post heat treatment (Table 2 and Figure 5D) indicated germ cell loss might be a potential reason partly responsible for the low testes weight. The stable concentration of mammal testosterone was maintained by the hypothalamic - pituitary - Leydig cell (HPL) axis [24]. Elevated scrotal temperature induced up-regulation of testosterone in patients, which might contribute to attenuate testicular oxidative stress-mediated apoptosis [20]. The testosterone concentration in serum was reduced after 14 days of chronic body heat in boars [25]. In present study, the testosterone concentration in serum was fluctuated, accompanying with increased temperature in scrotal and body surface, indicating that elevation of whole body heat disturbed the functions of Leydig cells and testosterone concentration was determined by heat-treated intension and exposed term. The peak value of testosterone concentration at 4th day suggested that mice might promote HPL axis to protect germ cells against heat-induced damage. However, chronic heat failed to maintain persistent activation of HPL axis and might inhibited the function of HPL axis after 12 days of whole body heat.

**Figure 7:**
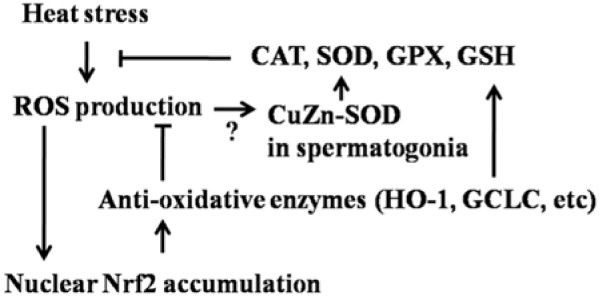
**Proposed model of Nrf2-mediated protection to whole body heat stress in mice testes.** ROS production induced by heat stress was inhibited by the antioxidative system in cytoplasm. Increased ROS stimulated Nrf2-antioxidant system and up-regulated CuZn-SOD concentration in spermatogonia. Thereby, the antioxidative system was enhanced and efficiently protected testes against oxidative damage.

Elevated testicular temperature induced oxidative stress, thereby resulting in apoptosis and germ cell death [12,20]. Testicular heat induced oxidative stress mainly by mitochondria-derived reactive oxygen species (ROS) and lipid peroxidation of the cellular membrane [26]. The increased oxidative stress was characterized by the elevated generation of MDA and increased expression of antioxidants [12,27]. During consecutive 4-day heat treatment in this study, both significant increased GSH amount and CAT activity implied the occurrence of heat-induced oxidative stress in testes (Figure 4B and 4D). However, both the unchanged activities of GPX, T-SOD and CnZn-SOD and MDA concentration suggested that the increased oxidative stress had not yet impaired membrane systems. As increased intension of heat treatment, the significant increased MDA concentration and down-regulation of GSH amount and the activities of CAT and GPX implied severe damage in testes. In addition, the most spermatids loss indicated heat-induced oxidative stress mainly damaged developing spermatids even though other cells were still in intact morphology. As a primary antioxidant enzyme, SOD plays an important role in the male testes [28]. A recent study revealed it's the high levels of CuZn-SOD and Zn that made spermatogonia more resistant to ROS and refrain from oxidative stress [29]. In this study, significant increased activities of CuZn-SOD and T-SOD on day 12 might result from high free radicals which triggered their expression in spermatogonia to scavenge the increased ROS. Consequently, the high SOD activity enhanced antioxidant system to protect spermatogonia against oxidative stress in mice testes (Figure 7).

Nrf2-antioxidant system has been already recognized as a prime molecular target against oxidative and electrophilic stresses via accumulating in nucleus and inducing expression of cytoprotective enzymes and related proteins [30]. Previous studies have reported that Nrf2-knockout mouse had high testicular and epididymal lipid peroxidation levels and low antioxidants levels which resulted in lower epididymal sperm motility than wild-type males [13]. In this study, it's obvious that 2-day consecutive heat stress induced Nrf2 accumulation in the nuclei of Leydig cells and germ cells. Our findings are now well documented that a strong nuclear Nrf2 accumulation exists in mouse testes. However, the decreased nuclear Nrf2 accumulation on day 12 in heated testes suggested Nrf2 nuclear export was inhibited even a high Nrf2 expression in cytoplasm. A recent study indicated failure to Nrf2-nuclear export, mediated by exportin-1, might be prevented by the failure to phosphorylate Tyr568 during oxidative stress [31]. Moreover, there are a variety of transcription factors antagonizing the Nrf2 activity such as nuclear factor-κB (NF-κB), peroxisome proliferator-activated receptor (PPAR), short form estrogen-related receptor β, estrogen receptor α and so on [32]. Therefore, decreased nuclear Nrf2 accumulation might be attributed to other involved pathways and impaired nuclear export caused by heat stress. To confirm activation of the Nrf2-ARE system in the present study, we measured the mRNA expression of *Nrf2* gene and three antioxidant genes containing ARE at their promoter regions (*Nrf2*, contains two ARE sequences [33]; *HO-1*, a well-characterized Nrf2 target gene [34]; *NQO1*, reduces quinones to hydroquinones to protect against oxidative stress [35]; *GCLC*, combines Glu and Cys as the first step in GSH production [36]). Previous studies have been reported that the activation of Nrf2 induced increased expression of Nrf2, HO-1, NQO1 and GCLC [37,38]. Consistent with the results above, our founding showed an increase in mRNA expression of *Nrf2*, *HO-1*and *GCLC* during consecutive heat stress which may reversely promote the expression of oxidative-mediated antioxidant genes via activation of Nrf2 transcription factor (Figure 7).

## Conclusions

In conclusion, the present study demonstrates that the whole body heat treatment triggers oxidative stress in mice testes, which acts as partners to induce cell death and reduced relative weight of testis. Nrf2 and its attended anti-oxidative enzyme systems are obviously up-regulated in the process of heat-mediated oxidative stress.

## Competing interests

The authors declare that they have no competing interests.

## Authors’ contributions

All authors participated in the design, interpretation of the studies, and review of the manuscript; YL performed data analysis and wrote the manuscript. All authors read and approved the final manuscript.
